# AM-MSFF: A Pest Recognition Network Based on Attention Mechanism and Multi-Scale Feature Fusion

**DOI:** 10.3390/e26050431

**Published:** 2024-05-20

**Authors:** Meng Zhang, Wenzhong Yang, Danny Chen, Chenghao Fu, Fuyuan Wei

**Affiliations:** 1 School of Computer Science and Technology, Xinjiang University, Urumqi 830017, China; menka@stu.xju.edu.cn (M.Z.); 107552103626@stu.xju.edu.cn (C.F.); wfy@stu.xju.edu.cn (F.W.); 2 Xinjiang Key Laboratory of Multilingual Information Technology, Xinjiang University, Urumqi 830017, China

**Keywords:** pest recognition, attention mechanism, multi-scale feature fusion, cross-entropy loss

## Abstract

Traditional methods for pest recognition have certain limitations in addressing the challenges posed by diverse pest species, varying sizes, diverse morphologies, and complex field backgrounds, resulting in a lower recognition accuracy. To overcome these limitations, this paper proposes a novel pest recognition method based on attention mechanism and multi-scale feature fusion (AM-MSFF). By combining the advantages of attention mechanism and multi-scale feature fusion, this method significantly improves the accuracy of pest recognition. Firstly, we introduce the relation-aware global attention (RGA) module to adaptively adjust the feature weights of each position, thereby focusing more on the regions relevant to pests and reducing the background interference. Then, we propose the multi-scale feature fusion (MSFF) module to fuse feature maps from different scales, which better captures the subtle differences and the overall shape features in pest images. Moreover, we introduce generalized-mean pooling (GeMP) to more accurately extract feature information from pest images and better distinguish different pest categories. In terms of the loss function, this study proposes an improved focal loss (FL), known as balanced focal loss (BFL), as a replacement for cross-entropy loss. This improvement aims to address the common issue of class imbalance in pest datasets, thereby enhancing the recognition accuracy of pest identification models. To evaluate the performance of the AM-MSFF model, we conduct experiments on two publicly available pest datasets (IP102 and D0). Extensive experiments demonstrate that our proposed AM-MSFF outperforms most state-of-the-art methods. On the IP102 dataset, the accuracy reaches 72.64%, while on the D0 dataset, it reaches 99.05%.

## 1. Introduction

Agriculture plays a crucial role in ensuring food security, promoting economic development, and maintaining ecological balance [1]. However, pests are one of the primary factors limiting agricultural development [2]. Traditionally, the early identification of pests relied heavily on agricultural experts. However, this approach was labor-intensive and lacked real-time capabilities [3]. With advancements in computer vision technology, automated pest recognition based on pest images has gained attention from researchers. Automated systems utilize computer vision techniques to analyze and interpret pest images, enabling farmers and agricultural practitioners to quickly and accurately identify specific pests that negatively impact crops. This technology reduces reliance on human experts and provides real-time pest detection capabilities, facilitating timely intervention measures and targeted pest control methods [4].

In insect identification tasks, extracting useful features from images faces several challenges due to the high diversity of pest species, as well as variability in their sizes and shapes. Past studies predominantly employed traditional machine learning methods using manually designed features, such as GIST [5], HOG [6], SIFT [7], and SURF [8]. However, these handcrafted features have limitations in capturing the large-scale variations in the shapes of target objects.

In recent years, deep learning has achieved robust feature learning and demonstrated state-of-the-art performance in various image classification tasks. Consequently, deep learning models based on convolutional neural networks (CNNs) have been widely applied in various image classification tasks in the agricultural domain, such as crop disease identification, crop classification, weed detection, and crop pest identification [9]. However, current datasets on pests are still very limited, with most datasets containing samples of only a few insect species. In addition, these datasets consist mainly of images of pests collected under controlled laboratory conditions. [3,9]. This limitation hampers the ability of deep learning models to perform insect pest recognition in real field conditions. Additionally, different insect pest species may have highly similar appearances, and there are also differences between the various forms of the same species (such as eggs, larvae, pupae, and adults) [10,11]. This implies that insect pest recognition tasks face challenges of significant intra-class variation and high inter-class similarity.

This paper proposes an identification network based on attention mechanism and multi-scale feature fusion (AM-MSFF) to address challenges such as complex backgrounds, large intra-class differences, small inter-class differences, and uneven data distribution in pest recognition. Our contributions are summarized as follows:

- The introduction of relation-aware global attention (RGA) helps the model focus on the pest part, suppresses interference from complex backgrounds, and enhance the model’s attention to pests;
- We propose the multi-scale feature fusion (MSFF) module, which extracts features at different scales and integrates these features to capture both the characteristics and contextual information of pests across different scales. This enables the model to better adapt to variations in the morphology and appearance of different pests. Additionally, we introduce generalized-mean pooling (GeMP) to better preserve important features and enhance the sensitivity to detailed information;
- An improved version of the cross-entropy loss function, called balanced focal loss (BFL), is proposed based on the focal loss (FL). BFL takes into consideration the number of samples for each class and adjusts the weights for each class accordingly. This adjustment allows the model to pay more attention to minority samples and hard-to-classify samples, thereby allowing the model to better handle class imbalance situations.

## 2. Related Work

With the advancement of computer vision technology, pest recognition methods have been continuously improved and innovated upon. Based on the approach of feature extraction, these methods can be broadly categorized into two types: traditional handcrafted feature-based methods and deep feature-based methods.

### 2.1. Handcrafted Features

In previous research, the process of feature extraction and classification required manual intervention, where researchers had to manually segment the insects of interest from the background. For instance, Mayo et al. [12] employed the image processing tool ImageJ (can be accessed at: https://imagej.net/ij/) [13] for feature extraction from insect images and utilized a support vector machine (SVM) for classification. Although this method successfully achieved automatic species identification of live specimens in the field without manually specifying regions of interest, it still required the segmentation of insects and background when the image background was highly cluttered. Yalcin [14] separated insects from the background using background subtraction and active contour models, extracting the outer boundaries of insects. Subsequently, they extracted features using Hu moments, elliptic Fourier descriptors (EFD), radial distance function (RDF), and local binary patterns (LBP), finding that LBP features performed better in terms of performance. Venugoban et al. [15] utilized ther histogram of oriented gradients (HOG) and speeded-up robust features (SURF) for image feature extraction, making full use of their ability to capture characteristics of local shape edges or gradient structures. They employed SVM for the multi-class classification of feature histograms. Xie et al. [16] employed sparse coding histograms of multiple feature modalities, combining multiple features of insect images for feature extraction. This method effectively quantifies original features such as color, shape, and texture, significantly enhancing recognition performance.

Handcrafted feature methods typically rely on raw image patches or manually designed image features, making them very sensitive to noise and background interference in natural images. They also struggle to adapt to the variations in the appearance of the same insect species at different stages. Furthermore, these methods often fail to capture mid-level and high-level features in insect appearances, and they also pose a significant computational burden. To address these issues, there is a need to develop more robust and discriminative feature descriptors that can automatically extract relevant information from insect appearances and adapt to changes in appearance across different insect species.

### 2.2. Deep Features

In recent years, CNNs such as ResNet [17] and GoogleNet [18] have achieved significant advancements in image classification tasks, garnering widespread attention. Consequently, an increasing number of researchers are exploring and adopting CNNs to address insect pest recognition problems. Li et al. [19] utilized CNNs to extract feature vectors from images and employed triplet loss training to distinguish between different insect pest species, ensuring the stable and reliable performance of the recognition system under various circumstances. Cheng et al. [20] introduced deep residual learning to overcome the problem of network degradation. Through optimization with deep residual learning, their method significantly improved the accuracy of insect pest image recognition in complex agricultural field backgrounds compared to simple CNNs like AlexNet [21]. Liu et al. [22] proposed the deep feature fusion residual network (DFF-ResNet), which enhances the model’s generalization ability by introducing feature fusion residual blocks that merge features from the previous layer with convolutional layers in the residual signal branch. Coulibaly et al. [23] introduced a crop pest recognition and localization network based on an interpretable approach, selecting inception-v3 as the backbone for feature extraction and highlighting the captured colors and shapes through visualized graphs. A combination of various interpretability methods better explains the reasoning process of deep learning systems and determines the optimal number of feature extraction layers. Hu et al. [24] proposed an insect recognition network based on a multi-scale dual-branch GAN-ResNet, utilizing ConvNeXt residual blocks to adjust computational scale and constructing a dual-branch structure to capture insect features of different sizes in input images while effectively extracting subtle features.

CNN models can automatically extract rich spatial and semantic information from images without the need for manual feature extraction, thereby reducing the workload of human involvement. However, CNN models rely heavily on large-scale data and are prone to recognition errors when dealing with complex backgrounds and lighting variations. Therefore, there is still room for improvement in this field, and further exploration is needed on how to enhance the model’s adaptability to complex backgrounds and lighting variations.

## 3. Proposed Method

**Figure 1:**
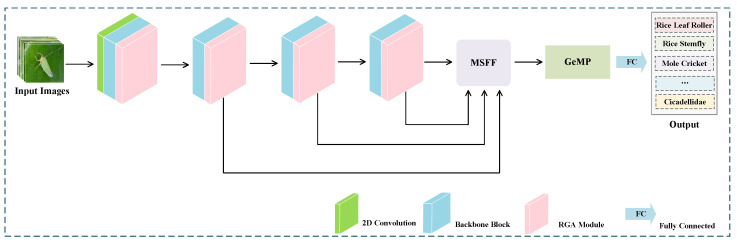
The structure of AM-MSFF.

As shown in Figure 1, the proposed AM-MSFF is based on the architecture of ResNet-50 [17] pre-trained on the ImageNet dataset. The network consists of the RGA module, MSFF module, and GeMP module, along with an improved focal loss [25] function called balanced focal loss (BFL). In the specific network structure, the RGA module models relationships between different positions in the image and weights them using attention mechanisms. The MSFF module fuses multi-scale feature information, focusing on both details and global features. The GeMP module better preserves the spatial information within feature maps. BFL adjusts the sample weights to balance the influence between different classes.

### 3.1. Relation-Aware Global Attention

Although the relation-aware global attention (RGA) module [26] was initially designed to address the problem of person re-identification, we can draw inspiration from its design principles and incorporate it into pest-related tasks. We combine the RGA module with deep residual networks to construct a feature extraction network based on relation-aware global attention. By learning the relationships between feature nodes and computing attention weights, the network can effectively explore discriminative regional features.

The RGA module weights input features through two subsidiary modules: the spatial relation-aware attention (RGA-S) submodule and the channel relation-aware attention (RGA-C) submodule. Firstly, the RGA-S submodule emphasizes the critical spatial information by analyzing the spatial relationships among input features and subsequently weighting the original features based on the learned weights. Subsequently, the RGA-C submodule further processes the weighted output from RGA-S by leveraging channel relationships and highlighting important channel information. This two-stage attention mechanism enables the model to more accurately focus on essential input feature information, thereby enhancing the model’s representation learning capability and performance.

**Figure 2:**
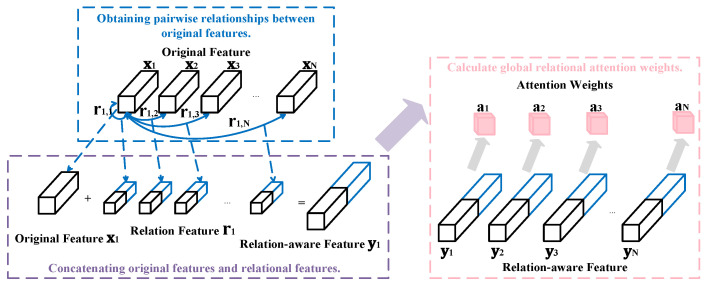
The structure of relation-aware global attention.

The RGA module’s structure, as shown in Figure 2, involves processing the input image through a frontend network to produce a feature map. Each feature vector in the feature map is represented as a feature node $x_{i}$, where i=1,2,…,N; *N* denotes the number of feature nodes. For each feature node $x_{i}$, its correlation with all other nodes $x_{j}$ (j=1,2,…,N) is computed, resulting in correlation values $r_{\left(i,j\right)}$ and $r_{\left(j,i\right)}$. The relationship vector for feature node $x_{i}$ is represented as $r_{i}=\left[r_{\left(i,1\right)},r_{\left(i,2\right)},\ldots ,r_{\left(i,N\right)},r_{\left(1,i\right)},r_{\left(2,i\right)},\ldots ,r_{\left(N,i\right)}\right]$. Subsequently, feature node $x_{i}$ and its relationship vector $r_{i}$ are concatenated to obtain the relation-aware feature $E_{i}$. Then, the attention weight $a_{i}$ for the current feature node is computed.

#### 3.1.1. Spatial Relation-Aware Global Attention

**Figure 3:**
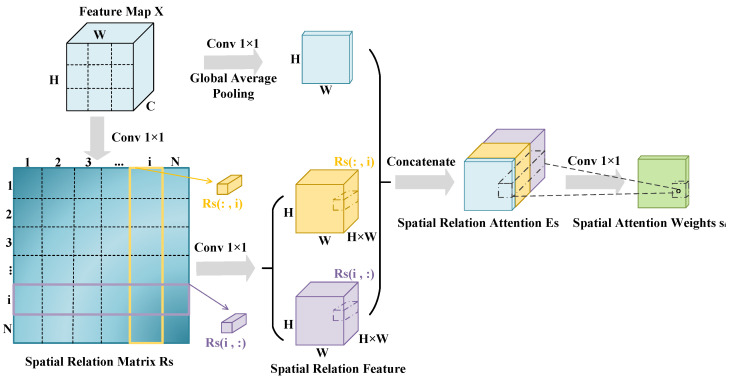
The structure of spatial relation-aware global attention.

RGA-S is a method for learning each feature node in the spatial dimension of the feature map. It compactly represents the pairwise relationships between all feature nodes and extracts structural information with a global context, as illustrated in Figure 3. Our approach incorporates RGA-S into the ResNet-50 network to learn the correlations between all feature nodes in the spatial dimension of the feature map, enabling the network to better focus on important spatial positions and feature nodes.

Specifically, for the input feature map $X\in R^{C\times H\times W}$ obtained from ResNet-50, each *C*-dimensional feature vector at every spatial position is regarded as a feature node. These nodes construct a node graph $G_{s}$, consisting of a total of N=W×H nodes. Each feature node is represented as $x_{i}$, where i=1,2,…,N. By performing a dot product operation, we can obtain the correlation $r_{\left(i,j\right)}$ between feature nodes $x_{i}$ and $x_{j}$, which can be defined by Equation (1):

(1) $$\left.\left\{\begin{array}{cc} r_{i,j} & =f_{s}\left(x_{i},x_{j}\right)=\theta_{s}{\left(x_{i}\right)}^{T}\phi_{s}\left(x_{j}\right)\\ \theta_{s}\left(x_{i}\right) & =\left(ReLU\left(BN(Con\nu \left(x_{i}\right))\right)\right)\\ \phi_{s}\left(x_{j}\right) & =\left(ReLU\left(BN\left(Con\nu (x_{j})\right)\right)\right) \end{array}\right.\right.$$

Here, the function $f_{s}$ represents the dot product operation, $\theta_{s}$ and $\phi_{s}$ are two embedding functions, BN stands for batch normalization, ReLU denotes the rectified linear unit activation function, and Conv represents 1×1 convolution operation. Similarly, the pairwise relationship between node *j* and node *i* is denoted as $r_{j,i}=f_{s}\left(x_{j},x_{i}\right)$, and $\left(r_{i,j},r_{j,i}\right)$ represents the bidirectional relationship between $x_{i}$ and $x_{j}$. Finally, the correlations between all nodes can be represented by the relation matrix $R_{S}\in R^{N\times N}$, where $r_{i,j}=R_{S}\left(i,j\right)$.

For the *i*-th feature node, the pairwise relationships with all nodes are stacked in a certain order to obtain a spatial relation vector $r_{i}=\left[R_{S}\left(i,:\right),R_{S}\left(:,i\right)\right]\in R^{2N}$. Then, the spatial relation vector is concatenated with the original feature information to incorporate both the global structural information and local original information, resulting in spatial relation attention $E_{S}$, which can be defined as Equation (2):

(2) Es=C(xi,ri)=(poolC(ψS(xi)),δS(ri))ψS(xi)=ReLUBN(Conv(xi))δS(ri)=ReLUBN(Conv(ri))

where $\psi_{S}$ and $\delta_{S}$ represent operations on the original features and spatial relation features, respectively. C denotes concatenation operation, $pool_{C}$ denotes global average pooling (GAP) operation along the channel dimension, and Conv reduces the channel dimension to one.

Through spatial relation-aware attention $E_{s}$, the attention weight value $s_{i}$ is computed for each position. This attention weight is then multiplied with the original features to obtain the intermediate feature $Y_{S}$ weighted by spatial relation-aware attention. The computation process is depicted in Equations (3) and (4).

(3) si=sigmoid(BN(Conv2(ReLU(BN(Conv1(Es))))))

(4) $$Y_{S}=\sum_{i=1}^{N}s_{i}\cdot x_{i}$$

where sigmoid represents the sigmoid activation function, $Conv_{2}$ reduces the number of channels to one, and $Conv_{1}$ reduces the dimensionality by a fixed ratio.

#### 3.1.2. Channel Relation-Aware Global Attention

**Figure 4:**
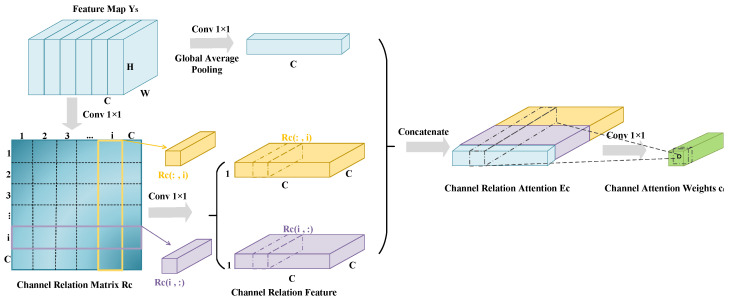
The structure of channel relation-aware global attention.

RGA-C learns various feature nodes along the channel dimension, compactly representing pairwise relationships among all feature nodes to obtain global structural information along the channel dimension, as illustrated in Figure 4. The approach in this paper incorporates RGA-C into the ResNet-50 network, enabling the learning of the correlations among all feature nodes in the channel dimension. This allocates different weights to each channel, enhancing the network’s focus on different channel information in pest images.

Specifically, the intermediate feature $Y_{S}$ obtained from the RGA-S submodule is used as the input feature for the RGA-C submodule. For the obtained feature map $Y_{S}\in R^{C\times H\times W}$, each feature map on every channel is considered a feature node, forming a graph $G_{C}$ with a total of *C* nodes. Each feature map on a channel is regarded as a feature node denoted as $y_{i}$, where i=1,2,…,C. The input feature $Y_{S}$ is compressed into $Y_{S}^{\prime }\in R^{(HW)\times C\times 1}$, and then transformed using two 1 × 1 convolutions to obtain two feature node vectors that are dot-producted to form the channel relation matrix $R_{C}\in R^{C\times C}$. The element $r_{\left(i,j\right)}$ of $R_{C}$ represents the pairwise relationship between node *i* and node *j*, defined by Equation (5):

(5) $$\left.\left\{\begin{array}{cc} r_{i,j} & =f_{c}\left(x_{i},x_{j}\right)=\theta_{C}^{T}\left(x_{i}\right)\varphi_{C}\left(x_{j}\right)\\ \theta_{C}^{T}\left(x_{i}\right) & =\left(ReLU\left(BN(Con\nu \left(x_{i}\right))\right)\right)\\ \varphi_{C}\left(x_{j}\right) & =\left(ReLU\left(BN\left(Con\nu (x_{j})\right)\right)\right) \end{array}\right.\right.$$

where $f_{c}$ represents dot product operation. Similarly, the correlation $r_{j,i}$ between feature nodes $x_{j}$ and $x_{i}$ can be obtained. The pairwise relationships between all nodes are represented by the matrix $R_{C}\in R^{C\times C}$. Stacking the relationships of the *i*th feature node with all nodes, we obtain the channel relation vector $r_{i}=\left[R_{C}\left(i,:\right),R_{C}\left(:,i\right)\right]\in R^{2N}$. Similar to Equation (3), we can obtain the final channel attention weight $c_{i}$. The attention weights are multiplied by the intermediate feature $Y_{S}$ to obtain the final output feature representation Y. The calculation process is shown in Equation (6):

(6) $$Y=\sum_{i=1}^{C}c_{i}\cdot y_{i}$$

### 3.2. Multi-Scale Feature Fusion

**Figure 5:**
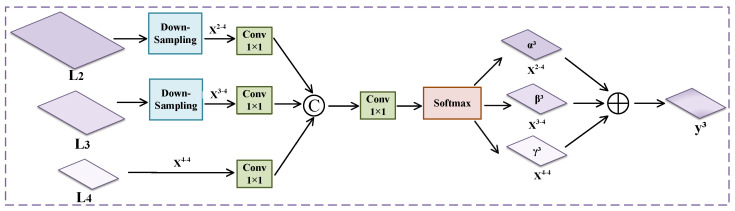
The structure of multi-scale feature fusion.

The purpose of adaptive spatial feature fusion (ASFF) [27] is to address the consistency issue among feature pyramids in object detection. By filtering conflicting information spatially, ASFF can weaken the inconsistency between features at different scales, thereby improving the scale invariance of features. Pest objects in pest images often vary in size and shape and may exhibit different detail and texture features. Therefore, relying solely on features from a single scale may not fully capture all useful information. Hence, inspired by the idea of ASFF, we propose multi-scale feature fusion (MSFF) to extract rich detail and global information from feature maps at different scales. The structure of MSFF is illustrated in Figure 5, and implementing MSFF involves two steps: feature scale adjustment and adaptive fusion.

Specifically, L2, L3, and L4 are different levels of the ResNet-50 model with attention mechanism, representing features at different scales. They, respectively, represent features at different scales. First, the sizes of the L2 and L3 feature layers are adjusted to match the size of the L4 feature map. $X^{2–4}$ represents resizing the L2 feature size to match the size of the L4 feature, while $X^{3–4}$ represents resizing the L3 feature size to match the size of the L4 feature. The L4 feature layer is denoted as $X^{4–4}$. Then, each layer’s channel number is compressed to eight through 1×1 convolution layers, concatenated along the channel dimension, followed by another 1 × 1 convolution to obtain a 3-channel weight used for weighted fusion of different level feature maps. Using the softmax operation, the fusion weight coefficients are bounded between [0,1], result in fusion coefficients $\alpha^{3}$, $\beta^{3}$, and $\gamma^{3}$ for L2, L3, and L4, respectively. The input feature maps are then weighted and fused using these coefficients to obtain the fused feature map $y^{3}$. Finally, the fused feature map is convolved with a 3 × 3 kernel with a stride of one to extract higher-level feature representations, enhancing the expressiveness and discriminability of the features. Therefore, the fused feature layer corresponding to MSFF is represented as shown in Equation (7).

(7) $$y^{3}=\alpha^{3}\cdot X^{2–4}+\beta^{3}\cdot X^{3–4}+\gamma^{3}\cdot X^{4–4}$$

where $\alpha^{3}+\beta^{3}+\gamma^{3}=1$, $\alpha^{3}$, $\beta^{3}$, and $\gamma^{3}$ are in the range [0,1].

MSFF effectively integrates information from different feature layers by adaptively adjusting their fusion ratios. Thus, MSFF dynamically adjusts the weights of features based on the importance of different parts of the image, enabling a more accurate capture of pest-related information present in the image. Through this approach, MSFF efficiently filters out conflicting information in the image, thereby improving the model’s accuracy in identifying pests.

### 3.3. Generalized-Mean Pooling

Traditional max pooling or average pooling methods are ineffective at capturing salient features with domain specificity. To address this issue, we introduce a learnable pooling layer known as generalized-mean pooling (GeMP) [28]. GeMP applies element-wise power operation to the input features followed by averaging, thereby better capturing important features with subtle differences in pest recognition tasks. Thus, through GeMP, the model can better understand detailed information in pest images and differentiate between different types of pests. Mathematically, GeMP can be represented by Equation (8):

(8) $$f={\left[f_{1}\cdots f_{k}\cdots f_{K}\right]}^{T},f_{k}=\left(\frac{1}{|X_{k}|}\sum_{x_{i}\in X_{k}}{\left.x_{i}^{p_{k}}\right)}^{\frac{1}{p_{k}}}\right.$$

where $f_{k}$ represents the output feature map, *K* is the number of feature maps in the last layer. *X* is the input feature map, $X\in R^{H\times W\times C}$, and $X_{k}\in R^{H\times W}$. $p_{k}$ is a pooling hyperparameter whose value is learned during backpropagation.

It is worth noting that when $p_{k}=1$, GeMP degenerates into average pooling, as shown in Equation (9):

(9) $$f={\left[f_{1}\cdot \cdot \cdot f_{k}\cdot \cdot \cdot f_{K}\right]}^{T},f_{k}=\frac{1}{|X_{k}|}\sum_{x_{i}\in X_{k}}x$$

when $p_{k}=\infty$, GeMP degenerates into max pooling, as shown in Equation (10):

(10) $$f={\left[f_{1}\cdots f_{k}\cdots f_{K}\right]}^{T},f_{k}=\max_{x_{i}\in X_{k}}x$$

### 3.4. Balanced Focal Loss

In pest recognition tasks, difficult samples are a key factor leading to inefficient model learning. The existence of difficult samples mainly stems from the imbalance in the number of pest categories and the low discriminability of small individual features. To address this issue, this study introduces focal loss to improve the problem.

Focal loss (FL) [25] is achieved by adding the modulation factor ${\left(1-p_{t}\right)}^{\gamma }$ on the basis of standard cross-entropy loss (CEL), which adaptively adjusts the contribution of samples to the loss based on their prediction accuracy. This helps the model to focus more on the misclassified and difficult samples. Specifically, the model reduces its attention on samples that can be predicted very accurately, as these samples already have good classification capability and do not significantly affect the model’s classification ability. For samples that are not predicted accurately or even predicted incorrectly, the model increases its attention, thereby improving its prediction capability for these samples and enhancing the overall performance. This design ensures that even if there are many easily classifiable samples, they will not dominate the model’s training process. Mathematically, FL can be represented by Equation (11):

(11) $$FL\left(p_{t}\right)=-{\left(1-p_{t}\right)}^{\gamma }\log\left(p_{t}\right)$$

where $p_{t}$ is the predicted probability and γ is the focusing parameter. By adjusting the weights of positive and negative samples, the model pays more attention to difficult-to-classify samples. When a sample is correctly classified, $p_{t}$ approaches 1, and thus ${\left(1-p_{t}\right)}^{\gamma }$ approaches 0, resulting in a decrease in the loss term and reducing attention to easy samples. Conversely, when a sample is misclassified, $p_{t}$ approaches 0, making the loss term larger and increasing attention to difficult samples.

In practical applications, a variant of FL with the addition of the α balancing factor often yields better results. The FL variant with α is defined as follows in Equation (12):

(12) $$FL\left(p_{t}\right)=-\alpha {\left(1-p_{t}\right)}^{\gamma }\log\left(p_{t}\right)$$

In the original FL, α is a manually set balancing factor used to adjust the weights of easy and hard samples. However, this approach may not effectively adapt to changes in the dataset and the dynamic adjustment requirements during model training. Therefore, we propose the balanced focal loss (BFL), which calculates α in an adaptive manner. First, we calculate α adaptively based on the distribution of target categories. We count the occurrences of each class in the targets to obtain a histogram. Then, we divide the count of each class in the histogram by the total number of samples to calculate the frequency of each class. This gives us the frequency of each class occurrence. Next, to convert the frequency into α weight coefficients, we perform a normalization operation. Specifically, we divide the frequency of each class by 10 and then subtract the result from 1. The purpose of this operation is to adjust the frequency to a range such that the maximum frequency corresponds to α = 1. This way, we obtain the weight coefficients of each class relative to other classes, which are used to balance the differences in the number of samples between different classes. The definition of α is as follows in Equation (13):

(13) $$\left\{\begin{array}{c} f_{i}=\frac{n_{i}}{N}\\ \alpha_{i}=1-\left(\frac{f_{i}}{10}\right) \end{array}\right.$$

where $f_{i}$ represents the occurrence frequency of the *i*th category, $n_{i}$ represents the number of samples in the *i*th category, $\alpha_{i}$ represents the weight coefficient of the *i*th category, and *N* is the total number of samples.

## 4. Experiments

In this section, we compare AM-MSFF with relevant state-of-the-art methods and validate the effectiveness of the added modules through a series of ablation studies.

### 4.1. DataSets

**Figure 6:**
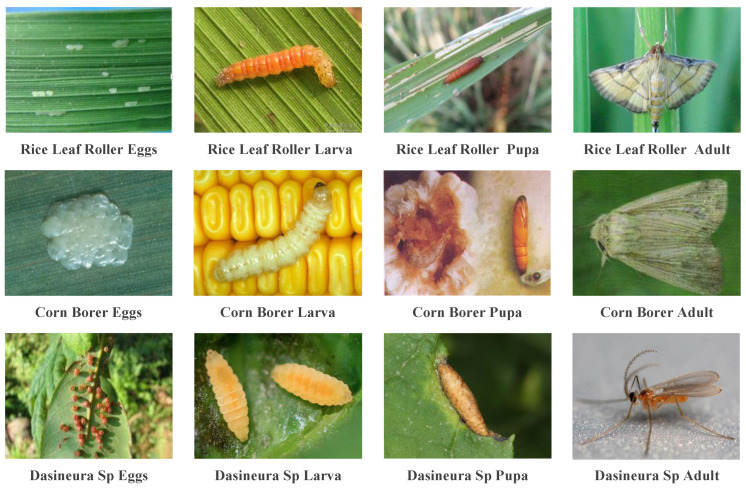
The example images from the IP102 dataset include various morphologies of insects, such as eggs, larvae, pupae, and adults.

The IP102 dataset [29] is currently the largest publicly available benchmark dataset for insects, covering eight crops including rice, corn, wheat, sugar beet, alfalfa, grapes, citrus, and mangoes. This dataset comprises a total of 75,222 insect images distributed across 102 categories, exhibiting a natural long-tailed distribution. Adopting a hierarchical classification approach, each insect is classified into a superclass reflecting its predation on crop types, along with subclasses labeled as pests damaging crops, encompassing images at different stages of insect development, such as egg, larva, pupa, and adult. Furthermore, insects at different growth stages may exhibit distinct appearance features. Additionally, different species of insects may share similar characteristics, further complicating insect classification. In our study, 45,095 images are used for training, 7508 for validation, and the remaining 22,169 for testing. The examples of the IP102 dataset are illustrated in Figure 6, and detailed information is provided in Table 1.

**Figure 7:**
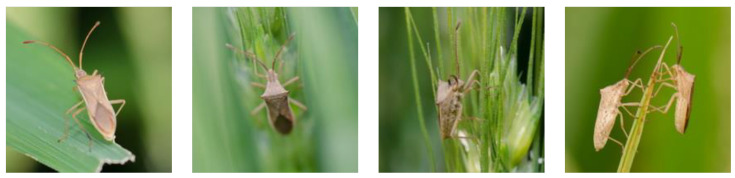
Example of Cletus punctiger (Dallas) in the D0 dataset.

The D0 dataset [30] consists of 4508 insect images with a resolution of 200×200 pixels, covering most of the common insect species found in several major field crops, including corn, soybeans, wheat, and rapeseed. In our study, we randomly divided D0 into three subsets, with 70% used for training. The remaining 30% was further divided, with 30% allocated for validation and the remaining 70% forming a new test set. Thus, in our research, 3155 images were used for training, 406 for validation, and the remaining 947 for testing. Table 1 lists the names of various insects along with their respective image counts. As indicated in the table, there is some degree of imbalance in the number of different insect species. Examples of the D0 dataset are shown in Figure 7, and detailed information is provided in Table 2.

### 4.2. Evaluation Metrics

Due to the class imbalance in both the IP102 and D0 datasets, we evaluate our proposed model using metrics such as macro average precision (MPre), macro average recall ($MRe_{c}$), macro average F1-score (MF1), accuracy (Acc), and geometric mean (GM). To equally weigh the importance of each class, we compute the recall for each class and then take their average to obtain $MRe_{c}$, as follows:

(14) $${\mathrm{Rec}}_{c}=\frac{{\mathrm{TP}}_{c}}{{\mathrm{TP}}_{c}+{\mathrm{FN}}_{c}}$$

(15) $${\mathrm{MRe}}_{c}=\frac{\sum_{c=1}^{C}{\mathrm{Rec}}_{c}}{C}$$

where *C* is the number of classes. $TP_{c}$ and $FN_{c}$ represent the true positives and false negatives for class *c*, respectively. Similarly, $Pre_{c}$ and MPre are calculated using the following formulas:

(16) $${\mathrm{Pre}}_{c}=\frac{{\mathrm{TP}}_{c}}{{\mathrm{TP}}_{c}+{\mathrm{FP}}_{c}}$$

(17) $$\mathrm{MPre}=\frac{\sum_{c=1}^{C}{\mathrm{Pre}}_{c}}{C}$$

where $FP_{c}$ represents the false positives for class *c*. MF1 is the harmonic mean of $MRe_{c}$ and MPre, calculated using the following formula:

(18) $$\mathrm{MF}1=2\frac{\mathrm{MPre}\cdot {\mathrm{MRe}}_{c}}{\mathrm{MPre}+{\mathrm{MRe}}_{c}}$$

Acc is calculated based on the true positive counts across all classes, computed as follows:

(19) $$\mathrm{Acc}=\frac{\mathrm{TP}}{N}$$

where *N* is the total number of samples. GM is computed based on the sensitivity of each class (represented as $S_{c}$), calculated as follows:

(20) $$S_{c}=\frac{{\mathrm{TP}}_{c}}{{\mathrm{TP}}_{c}+{\mathrm{FN}}_{c}}$$

(21) $$\mathrm{GM}=\prod_{c=1}^{c}\sqrt[{c}]{S_{c}}$$

GM is equal to 0 if and only if $S_{c}$ is equal to 0. To avoid this issue, we replace the values of sensitivity that are 0 with 0.001.

### 4.3. Experiment Settings

We conducted preprocessing steps on the input images, where the size of the image is h×w, with *h* and *w* representing the height and width of the image, respectively. Firstly, we resized the image to $h^{\prime }\times w^{\prime }$ to maintain the aspect ratio of the original image. We chose to adjust the smaller of *h* and *w* to 256 and adjusted the larger value based on the ratio of the larger to the smaller value. This helps maintain the aspect ratio of the image and adapts to the input requirements of the model. During the training phase, we applied random cropping as a data augmentation technique with a window size of 256×256 to address overfitting issues. Through random cropping, we randomly selected different sub-regions from the image, increasing the diversity and generalization capability of the data. In the testing phase, we used center cropping with the same size window as in the training phase. This ensures that the image region used in the testing phase is similar to that in the training phase, resulting in comparable results.

During the training process, we utilized ResNet-50 pre-trained on the ImageNet dataset as the backbone network, with BFL as the classification loss function. To optimize the model, we used the Adam optimizer with a learning rate initialized to $1\times 10^{-4}$, and coefficients $\beta_{1}$ and $\beta_{2}$ set to 0.9 and 0.999, respectively. To control the decay of the learning rate, we employed an exponential decay method with a decay rate of 0.96. We partitioned the training data into batches, with a batch size of 64 for the IP102 dataset and 32 for the D0 dataset, as it is smaller. We set the maximum number of training epochs to 100.

### 4.4. Experimental Results

To assess the effectiveness of our approach, we compared it with several state-of-the-art methods and conducted experiments on the IP102 and D0 datasets. The experimental results are shown in Table 3 and Table 4.

On the IP102 dataset, we compared AM-MSFF with ResNet-50 implemented in [29], as well as some variants of ResNet, namely, FR-ResNet [31] and DMF-ResNet [32]. The results indicate that AM-MSFF outperforms ResNet-50 and its variants. Additionally, our method demonstrates competitiveness when compared with other state-of-the-art models. Specifically, as shown in Table 3, the AM-MSFF method achieves state-of-the-art results in recognition accuracy, surpassing MMAL [34] by 0.48%. It ranks slightly lower than GAEnsemble [33] in MPre but still achieves an excellent second place. It is lower than MMAL by 1.76% in $MRe_{c}$ but maintains the second position. It is slightly lower than GAEnsemble by 0.14% in MF1 but outperforms MMAL by 3.04% in GM.

On the D0 dataset, we compared AM-MSFF with ResNet-50 implemented in [33] and other state-of-the-art methods, achieving the best results. Specifically, as shown in Table 4, compared with the currently best-performing GAEnsemble, AM-MSFF outperforms it by 0.24%, 0.04%, 0.05%, and 0.03% in terms of ACC, MPre, $MRe_{c}$, and MF1, respectively. This result further demonstrates that our model has higher accuracy and better generalization ability.

### 4.5. Ablative Study

To evaluate the performance of the AM-MSFF model, we conducted ablation experiments to analyze the contributions of its four components to the model’s performance. Since the recognition difficulty of the IP102 dataset is greater, we chose to conduct ablation experiments on this dataset, and the experimental results are presented in Table 5.

Our ResNet-50 model performs significantly better in terms of performance compared to the implementation in [29]. Our model adopts the random cropping augmentation technique, which randomly crops input images during the training process, thereby increasing the diversity and richness of the data. This helps improve the model’s generalization ability and robustness, making it better suited to different scenarios and variations. Additionally, we used the Adam optimizer, which is a gradient-based adaptive optimization algorithm. Compared to the stochastic gradient descent (SGD) optimizer used in [29], Adam converges faster and finds better local minima, thereby improving the efficiency and performance of the model training.

#### 4.5.1. The Impact of Relation-Aware Global Attention

To validate the impact of RGA on model performance, we conducted a series of ablation experiments, and the results are shown in Table 5. Firstly, compared to the baseline model, adding only the RGA module led to a performance improvement. Specifically, the model with the added RGA module showed an increase of 0.46% in ACC, 0.45% in MPre, 0.58% in $MRe_{c}$, 0.47% in MF1, and 0.13% in GM. Furthermore, compared to the model without the RGA module, the AM-MSFF model with the added RGA module exhibited significant improvements in all metrics. Specifically, the AM-MSFF model with the added RGA module showed an increase of 0.39% in ACC, 0.52% in MPre, 0.12% in $MRe_{c}$, 0.38% in MF1, and 0.51% in GM.

The results indicate that the introduction of the RGA module effectively enhances model performance. The RGA module improves the representation capacity of features related to pests and captures relationships between features, effectively enhancing the model’s ability to recognize pests.

#### 4.5.2. The Impact of Multi-Scale Feature Fusion

To validate the impact of MSFF on model performance, we conducted a series of ablation experiments, and the results are shown in Table 5. Firstly, compared to the baseline model, adding only the MSFF module led to a 0.33% increase in ACC, a 0.36% increase in MPre, a slight decrease of 0.17% in $MRe_{c}$, a 0.12% increase in MF1, and a 0.36% increase in GM. Although there was a slight decrease in $MRe_{c}$, the improvement in other performance metrics indicates its effectiveness in enhancing the model. Secondly, compared to the case where the MSFF module was not added in the AM-MSFF model, adding MSFF led to more significant improvements in all performance metrics. It resulted in a 0.39% increase in ACC, a 0.51% increase in MPre, a 0.12% increase in $MRe_{c}$, a 0.38% increase in MF1, and a 0.51% increase in GM.

The experimental results demonstrate that by adding the MSFF module, our model can fully utilize feature maps from different scales. By integrating feature information from different scales, the model can better understand and capture the details and features of pests comprehensively, enhancing its perception ability towards pest targets, and improving the accuracy of identification results.

#### 4.5.3. The Impact of Generalized-Mean Pooling

To assess the impact of GeMP on model performance, we conducted a set of comparative ablation experiments, and the experimental results are listed in Table 6. The results show that GeMP significantly improves model performance. Firstly, we compared the use of GeMP with global average pooling (GAP) and global max pooling (GMP) in the baseline model. The results indicate that compared to the model using GAP, the model using GeMP achieved an increase of 0.49% in ACC, 0.47% in MPre, 0.87% in $MRe_{c}$, 0.66% in MF1, and 0.49% in GM. Compared to the model using GMP, the model using GeMP achieved an increase of 0.18% in ACC, 0.05% in MPre, 0.46% in $MRe_{c}$, 0.30% in MF1, and 0.33% in GM.

Secondly, replacing GAP with GeMP in the AM-MSFF module significantly improved model performance. The experimental results show that compared to the model using GAP, the model using GeMP achieved an increase of 0.76% in ACC, 0.50% in MPre, 0.52% in $MRe_{c}$, 0.52% in MF1, and 0.34% in GM. Similarly, compared to the model using GMP in the AM-MSFF module, using GeMP also led to a significant improvement in model performance. The experimental results show that compared to the model using GMP, the model using GeMP achieved an increase of 0.66% in ACC, 1.21% in MPre, 0.70% in MF1, and 0.35% in GM. Although there is a slight decrease of 0.14% in $MRe_{c}$, the significant improvements in other performance metrics indicate the effectiveness of GeMP.

The experimental results indicate that GeMP achieves a higher recognition accuracy than GMP, and GMP outperforms GAP. In pest identification tasks, crucial information in feature maps tends to be concentrated in local regions, with other areas being relatively less important. GMP retains only the most significant parts of each feature map during pooling, whereas GAP simply averages the entire feature map, potentially leading to the loss or blurring of local information.

However, GeMP performs a weighted average based on the activation level of features, which more accurately reflects the crucial information in feature maps by considering the intensity of each feature during activation. In contrast, GAP cannot distinguish the importance of different features, and GMP overlooks other important activated information. Through GeMP, the model can more effectively utilize useful information in feature maps, thereby enhancing its perception and recognition accuracy of pest targets. Additionally, this weighted averaging approach helps the model better adapt to variations in different scenes and features, improving its generalization ability and robustness.

#### 4.5.4. The Impact of Balanced Focal Loss

To validate the impact of BFL on model performance, we conducted a series of ablation experiments, and the results are presented in Table 7. In the experiments, we defaulted to using CEL, and replacing CEL with BFL loss on the baseline led to improvements in multiple metrics. Specifically, ACC increased by 0.6%, MPre increased by 0.36%, $MRe_{c}$ increased by 0.37%, and MF1 increased by 0.31%. Although there was a slight decrease of 0.03% in GM, the improvements in other performance metrics suggest that BFL is effective in enhancing the model’s classification ability. When using BFL in the AM-MSFF model, compared to using FL, although there was a slight decrease in $MRe_{c}$ and MF1 metrics, the recognition accuracy was higher. Compared to using CEL, there was a 0.24% increase in ACC, a 0.09% increase in MPre, a 0.14% increase in $MRe_{c}$, a 0.39% increase in MF1, and a 0.09% increase in GM.

Since BFL assigns lower weights to easily classifiable samples and higher weights to difficult-to-classify samples during training, it may lead to a misclassification of some easily classifiable samples, thereby reducing MRe_c_ and MF1. However, compared to CEL, BFL still effectively improves the problem of imbalanced data distribution and enhances the model’s classification performance.

#### 4.5.5. Discussion of Results

From Table 3 and Table 4, it can be observed that the AM-MSFF method outperforms other state-of-the-art methods in terms of recognition accuracy on the IP102 and D0 datasets, demonstrating a higher performance. Additionally, in terms of evaluation metrics such as MPre, MRe_c_, MF1, and GM, the AM-MSFF method also demonstrates competitiveness comparable to other state-of-the-art models.

However, in Table 5, we notice that the “AM-MSFF without GeMP” shows a decrease in accuracy compared to the “Baseline + RGA”. After analysis, we believe that this result may be due to an imbalance in local–global information. Although the RGA module is designed as a global attention mechanism, it also tends to focus more on the relationships between local regions, potentially leading to the neglect of some important contextual information when processing global information. With the addition of the MSFF module, the model’s ability to utilize global information is enhanced, as MSFF can better integrate features at different scales. However, this may also result in the model overly focusing on local information, leading to an imbalance when processing global information. Therefore, despite the overall improvement in utilizing information by the model, the imbalance may lead to a decrease in accuracy. It is worth noting that although the model’s recognition accuracy decreases, there is a significant improvement in metrics such as MPre, MRe_c_, MF1, and GM compared to only adding a single RGA and MSFF module, indicating that the model is able to more accurately capture and express features at different scales.

### 4.6. Visualization

In this section, we use the Grad-CAM method [42] to visualize the attention regions of our proposed model in the input images, helping to interpret the model’s predictions and understand its behavior. Grad-CAM calculates the gradients of the target class to identify the feature map regions that play a crucial role in the final prediction and visualize them as class activation maps.

**Figure 8:**
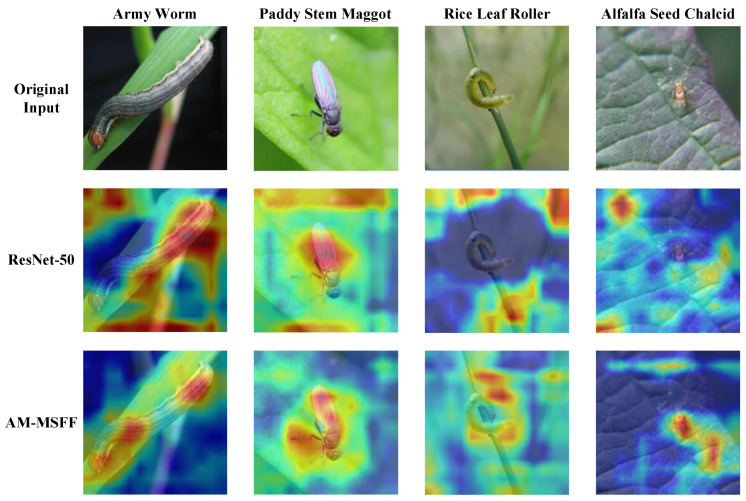
Visualization of Grad-CAMs produced by ResNet-50 and AM-MSFF.

Figure 8 displays the visual representation of the attention regions of the model in the input images. Even though insects like alfalfa seed chalcid are small, AM-MSFF is still able to focus on the insects in the input images. In contrast, although ResNet-50 can correctly focus on the insect’s location in most cases, it seems to prefer larger and less accurate regions for prediction, leading to relatively poorer performance.

## 5. Conclusions

This paper proposes a pest identification network based on attention mechanism and multi-scale feature fusion (AM-MSFF), which consists of three key modules: the RGA module, the MSFF module, and the GeMP module. Additionally, an improved loss function called BFL is proposed for the classification task. In the specific network architecture, the RGA module models the relationship between different positions in the image and weights them using an attention mechanism. This enables the network to focus on and highlight pest areas while suppressing interference from irrelevant regions. The MSFF module enhances the model’s perception and representation capabilities by fusing multi-scale feature information and paying attention to both details and global features. Unlike traditional GAP and max pooling, the GeMP module better preserves spatial information in the feature map, improving the perception of local details. In addition, to address the issue of class imbalance, this study proposes the use of BFL as a replacement for the cross-entropy loss to adjust sample weights. Experimental results on the IP102 and D0 datasets demonstrate the outstanding performance of the AM-MSFF method. On the IP102 dataset, the accuracy reaches 72.64%, while on the D0 dataset, it reaches 99.05%. Compared to other networks, the AM-MSFF method achieves high levels of accuracy.

In future research, on the one hand, we plan to delve into the characteristics and features of pest image data and design more targeted, efficient, and streamlined network architectures. On the other hand, we also aim to further enhance pest recognition performance through multimodal fusion. In addition to image data, pests may also be accompanied by other sensory data, such as sound and vibration. We can fuse these different modalities of data to obtain more comprehensive and accurate pest information.

## Figures and Tables

**Table 1:** The detailed information about the IP102 dataset.

Crop Type	Rice	Corn	Wheat	Beet	Alfalfa	Vitis	Citrus	Mango
Number of Categories	14	13	9	8	13	16	19	10
Number of Images	8417	14,004	3418	4420	10,390	17,551	7273	9738

**Table 2:** The detailed information about the D0 dataset.

No.	Name	Quantity	No.	Name	Quantity
1	Dolycoris baccarum (Linnaeus)	87	21	Stollia ventralis (Westwood)	72
2	Lycorma delicatula (White)	92	22	Nilaparvata lugens (Stål)	62
3	Eurydema dominulus (Scopoli)	150	23	Diostrombus politus Uhler	238
4	Pieris rapae (Linnaeus)	71	24	Phyllotreta striolata (Fabricius)	187
5	Halyomorpha halys (Stål)	101	25	Aulacophora indica (Gmelin)	78
6	Spilosoma obliqua (Walker)	66	26	Laodelphax striatellus (Fallén)	61
7	Graphosoma rubrolineata (Westwood)	116	27	Ceroplastes ceriferus (Anderson)	100
8	Luperomorpha suturalis Chen	101	28	Corythucha marmorata (Uhler)	98
9	Leptocorisa acuta (Thunberg)	133	29	Dryocosmus kuriphilus Yasumatsu	50
10	Sesamia inferens (Walker)	126	30	Porthesia taiwana Shiraki	141
11	Cicadella viridis (Linnaeus)	138	31	Chromatomyia horticola (Goureau)	114
12	Callitettix versicolor (Fabricius)	156	32	Iscadia inexacta (Walker)	79
13	Scotinophara lurida (Burmeister)	117	33	Plutella xylostella (Linnaeus)	69
14	Cletus punctiger (Dallas)	169	34	Empoasca flavescens (Fabricius)	133
15	Nezara viridula (Linnaeus)	175	35	Dolerus tritici Chu	88
16	Dicladispa armigera (Olivier)	150	36	Spodoptera litura (Fabricius)	130
17	Riptortus pedestris (Fabricius)	110	37	Corythucha ciliata (Say)	90
18	Maruca testulalis Geyer	73	38	Bemisia tabaci (Gennadius)	147
19	Chauliops fallax Scott	68	39	Ceutorhynchus asper Roelofs	146
20	Chilo suppressalis (Walker)	93	40	Strongyloides variegatus (Fairmaire)	135

**Table 3:** The comparison of classification performance on the IP102 dataset. Bold text indicates the best result, and underline is used to indicate the second-best result.

Model	ACC	MPre	MRe_c_	MF1	GM
ResNet-50 [29] (2019)	49.4	43.7	39.1	40.5	30.7
FR-ResNet [31] (2019)	55.24	-	-	54.18	-
DMF-ResNet [32] (2020)	59.22	-	-	58.37	-
GAEnsemble [33] (2020)	67.13	**67.17**	67.13	**65.76**	-
MMAL [34] (2021)	72.15	62.63	**69.13**	64.53	58.43
STN-SE-ResNet50 [35] (2021)	69.84	-	-	-	-
MobileNetV2 + Sparse + CutMix + DynamicLR [36] (2022)	71.32	-	-	-	-
ResNet152 + Vision-Transformer + Swin-Transformer [37] (2023)	65.6	60.9	59.7	60.3	-
GPA-Net [38] (2023)	56.9	45.9	43.8	45.0	-
AM-MSFF	**72.64**	64.54	67.37	65.62	**61.48**

**Table 4:** The comparison of classification performance on the D0 dataset. Bold text indicates the best result, and underline is used to indicate the second-best result.

Model	ACC	MPre	MRe_c_	MF1
MLLF + MKB [30] (2018)	89.3	-	-	-
CNNs [9] (2019)	95.97	-	-	-
GAEnsemble [33] (2020)	98.81	98.88	98.81	98.81
ResNet-50 [33] (2020)	92.18	92.74	92.18	92.07
ACEDSNet [39] (2022)	96.15	-	-	-
FcsNet [40] (2022)	98.33	98.49	98.33	98.34
SBPEnsemble [41] (2023)	96.18	96.45	95.37	-
AM-MSFF	**99.05**	**98.92**	**98.86**	**98.84**

**Table 5:** Ablation experiment results of RGA, GeMP, and MSFF on the IP102 dataset. Bold text indicates the best result.

Model	ACC	MPre	MRe_c_	MF1	GM
ResNet-50 (baseline)	71.30	63.46	65.24	64.12	60.64
baseline + RGA	71.76	63.91	65.82	64.59	60.77
baseline + GeMP	71.79	63.93	66.11	64.78	61.13
baseline + MSFF	71.63	63.82	65.07	64.24	61.00
AM-MSFF without MSFF	72.01	63.94	66.39	64.85	60.84
AM-MSFF without GeMP	71.64	63.95	65.99	64.71	61.01
AM-MSFF without RGA	72.01	63.93	66.39	64.85	60.84
AM-MSFF	**72.40**	**64.45**	**66.51**	**65.23**	**61.35**

**Table 6:** Comparative ablation experiment results of GAP, GMP, and GeMP on the ip102 dataset. Bold text indicates the best result.

Model	ACC	MPre	MRe_c_	MF1	GM
Baseline with GAP	71.30	63.46	65.24	64.12	60.64
Baseline with GMP	71.61	63.88	65.65	64.48	60.80
Baseline with GeMP	71.79	63.93	66.11	64.78	61.13
AM-MSFF with GAP	71.64	63.95	65.99	64.71	61.01
AM-MSFF with GMP	71.74	63.24	**66.65**	64.53	61.00
AM-MSFF with GeMP	**72.40**	**64.45**	66.51	**65.23**	**61.35**

**Table 7:** Loss function ablation experiment results on the IP102 Dataset. Bold text indicates the best result, and underline is used to indicate the second-best result.

Model	ACC	MPre	MRe_c_	MF1	GM
Baseline with CEL	71.30	63.46	65.24	64.12	60.64
Baseline with FL	71.84	63.73	66.33	64.74	60.85
Baseline with BFL	71.95	63.82	65.61	64.43	60.61
AM-MSFF with CEL	72.40	64.45	66.51	65.23	61.35
AM-MSFF with FL	72.61	**64.54**	**67.73**	**65.73**	61.39
AM-MSFF with BFL	**72.64**	**64.54**	67.37	65.62	**61.48**

## Data Availability

Data are contained within the article.
